# The outcome of ART in males with impaired spermatogenesis

**DOI:** 10.4103/0974-1208.44114

**Published:** 2008

**Authors:** VS Mangoli, SP Dandekar, SK Desai, RV Mangoli

**Affiliations:** IVF, Fertility Clinic and IVF Centre, 101-Shanti Niketan, V Gandhi Road, Gamdevi, Mumbai, Maharashtra, India; 1 Department of Biochemistry, GS Medical College, KEM Hospital, Parel, India; 2 IVF, Fertility Clinic and IVF Centre, 12-Spring Field, V Gandhi Road, Gamdevi, Mumbai, Maharashtra, India

**Keywords:** IUI, IVF, ICSI, male factor

## Abstract

**AIMS:**

This study was conducted to evaluate the outcomes of assisted reproductive technology (ART) procedures, viz., intrauterine insemination (IUI), in vitro fertilization (IVF) and intracytoplasmic sperm injection (ICSI) in males with impaired spermatogenesis.

**SETTINGS AND DESIGN:**

The subjects of the study were infertile couples who were undergoing ART treatment due to male factor indications. The project was designed to correlate the outcome of the ART treatment and its efficacy in different study groups.

**METHODS:**

Males were grouped as: 1. oligozoospermia (n = 153), 2. asthenoteratozoospermia (n = 158), 3. obstructive azoospermia (n = 110) and 4. nonobstructive azoospermia (n = 58). Patients from groups 1 and 2 were considered for IUI, IVF and ICSI. Those from group 3 were considered for IVF and ICSI and the 4th group underwent only ICSI treatment.

**RESULTS:**

Oligozoospermia showed lower pregnancy rates with IUI than with both IVF and ICSI. An average minimum native and postharvest count was obtained to get an acceptable IUI outcome. Asthenoteratozoospermia had the lowest pregnancy rate with IUI as compared to IVF, whereas ICSI showed significantly higher pregnancy rates in this group. Obstructive azoospermia showed significant improvement with ICSI over IVF. In nonobstructive azoospermia, ICSI resulted in a 27.58% pregnancy rate.

**CONCLUSION:**

The IUI outcome was impressive though less effective whereas there was no difference between the IVF and ICSI outcomes in oligozoospermia. In asthenoteratozoospermia, ICSI showed a significant advantage over IUI and IVF, with IUI resulting in poor outcome in this group. In obstructive azoospermia, ICSI had a distinct advantage over IVF whereas in nonobstructive azoospermia, ICSI, the only option, was found to be effective and helpful in achieving an acceptable pregnancy rate.

## INTRODUCTION

In male factor-associated infertility, often the decisions regarding the choice of treatment are taken on the basis of the native sperm count and morphology. Due to the lack of unanimously accepted criteria, treatment for male factor-associated infertility remains largely empirical. Such decisions may result in either complete fertilization failure or unnecessary manipulation of gametes for complicated and expensive modalities such as ICSI. This study was aimed to compare the efficacy of three most widely used treatment modalities for male factor-related infertility, *viz*., intrauterine insemination (IUI), *in vitro* fertilization (IVF) and intracytoplasmic sperm injection (ICSI).

## MATERIALS AND METHODS

Couples undergoing treatment for the sole cause of male factor-related infertility were considered as subjects for this study. Patients were divided into four study groups:

1. Oligozoospermia: Those with an average native count of < 20 million sperms/mL
2. Asthenoteratozoospermia: Those having < 30% motility with > 60% abnormal forms.
3. Obstructive azospermia: Sperms formed in the testis are unable to get ejaculated through semen mainly due to obstruction either in the epididymis, vas deferens or ejaculatory ducts.
4. Nonobstructive azospermia: Men with testicular failure having Sertoli cell-only pattern, maturation arrest or hypospermatogenesis on testis biopsy.

### Sperm retrieval

Depending upon the indication, sperms were obtained from semen samples from groups 1 and 2 whereas in group 3, they were retrieved by using the percutaneous, epididymal sperm aspiration (PESA) or microepididymal sperm aspiration (MESA) technique.[1,2] Sperms were retrieved from testicular biopsies from patients in group 4.[3] Eighty patients comprised group 4 of which no injectable sperms were obtained in 22 of these 80 cases (27.5%).

### Assisted reproductive procedures

Assisted reproductive procedures include intrauterine insemination (IUI), *in vitro* fertilization (IVF) and intracytoplasmic sperm injection (ICSI).

### Intrauterine insemination

IUI involves the manipulation of male gametes. Clinically, the female partner underwent controlled ovarian hyperstimulation (COH) using a standard protocol extensively described elsewhere.[4] When the leading follicle reached a size of 20 mm, the male partner's semen sample was collected in a sterile wide-mouthed container. A thorough semen analysis was done to evaluate the sperm count, motility, abnormal forms and forward progression. The liquefied semen sample was overlaid on 90, 70 and 40% density gradients made from a 100% stock solution (Sperm Grad, Vitrolife, Sweden). After centrifugation at 300 *g* for 20 min, the pellet was resuspended in 0.6–1 mL medium (sperm rinse, Vitrolife, Sweden) and a one-step swim-up was performed. If the initial count was < 5,000,000, the pellet was resuspended after centrifugation and directly used for the procedure. The suspension containing the spermatozoa was loaded into a soft, sterile catheter using a 1 mL tuberculin syringe and injected into the uterine cavity. The IUI procedure was performed for two days. An independent study was undertaken to correlate the native sperm count and pregnancy outcome in group I.

### *In vitro* fertilization

*In vitro* fertilization (IVF) and ICSI involve the manipulation of both male and female gametes outside the body. Patients underwent controlled ovarian hyperstimulation (COH) using a GnRH agonist along with a recombinant FSH protocol.[5] Ovum pick-up was done 36 h post hCG administration and these oocytes were cultured in tissue culture plastic petridishes (Falcon 3037) with insemination medium (G1, Vitrolife, Sweden). Semen samples were processed by using the density gradient technique as described above.

The processed sperms were dispersed around these oocytes at a concentration of 5000–10,000 sperms/oocyte/mL and the oocytes were observed for evidence of fertilization (the presence of two pronuclei) 16–20 h postinsemination. The zygotes were observed after 24 and 48 h for their development; usually, after 72 h of insemination, embryos reach the stage of forming 6–8 blastomeres The embryos were graded based on the number of blastomeres, their uniformity and evenness, the extent of fragmentation in and around the blastomeres, zona thickness and appearance of the ooplasm.[6] Three embryos were selected and transferred into the miduterine cavity.

### Intracytoplasmic sperm injection

Palermo *et al.* successfully applied this technique in humans in 1993 for enhancing fertilization, particularly, in cases of severe male factor indications.[7] The oocytes were collected as described above. After 2–4 hours, the cumulus surrounding the oocyte was removed using 80 IU/mL of hyaluronidase (Hyase, Medicult, Netherlands) and the denuded oocytes were kept in ICSI droplets containing G Mops medium (Vitrolife, Sweden). Sperms harvested from the male partner were deposited in a small polyvinylpyrrolidone (PVP) droplet (PVP, Medicult, Netherlands)—a viscous solution that restricts sperm movements. Using a micromanipulator attached to an inverted microscope, one morphologically normal spermatozoon was injected into the cytoplasm of the oocytes. Except for the insemination technique, all the other stages were the same as those for IVF. Statistical analysis was done by Student's independent t-test.

## RESULTS

**Figure 1:**
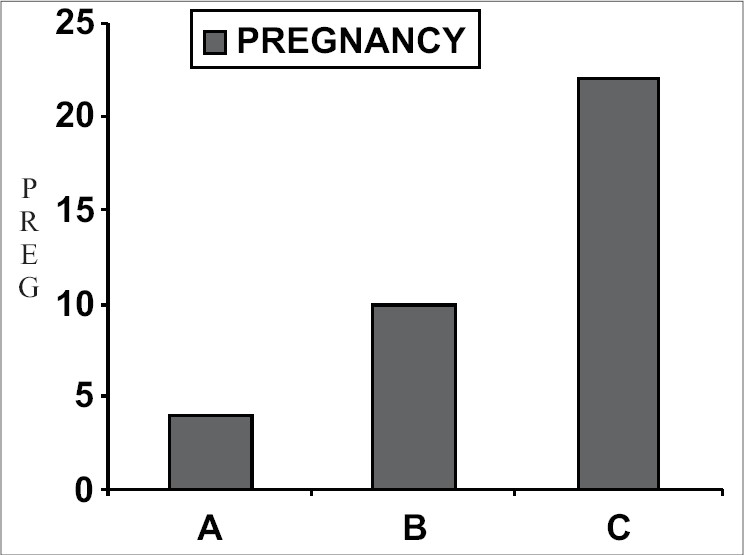
Native count and pregnancy outcome

Group I was divided into three categories wherein all the categories had a minimum 50% motility with initial grades of 1–2:

- Native count < 5 × 10^6^, n = 50, Pregnancy rate: 2 (4%)
- Native count = 5–15 × 10^6^, n = 50, Pregnancy rate: 5 (10%)
- Native count = 15–20 × 10^6^, n = 50, Pregnancy rate: 11 (22%) [Table 1] [Figure 1]

**Table 1:** Correlation between native count and intrauterine insemination outcome

Category (n = 50) (%)	Native count (× 106)	Pregnancy
A	<5	2(4%)
B	5–15	5(10%)*
C	15–20	11(22%)†

* *P* < 0.005

† *P* < 0.001

**Figure 2:**
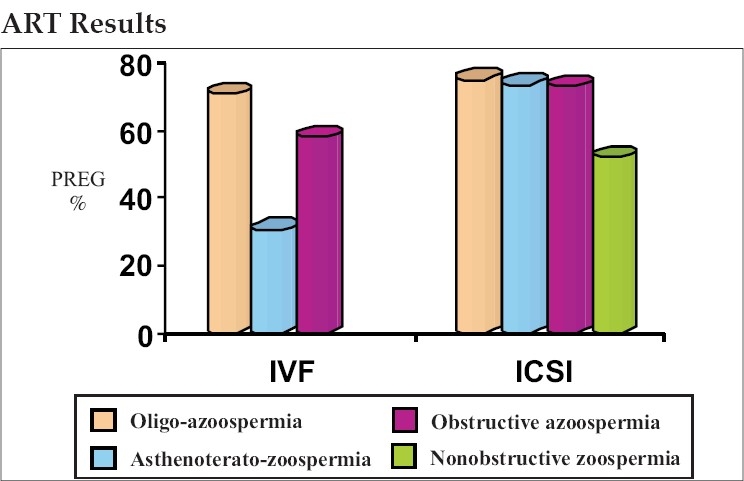
Fertilization outcome

**Figure 3:**
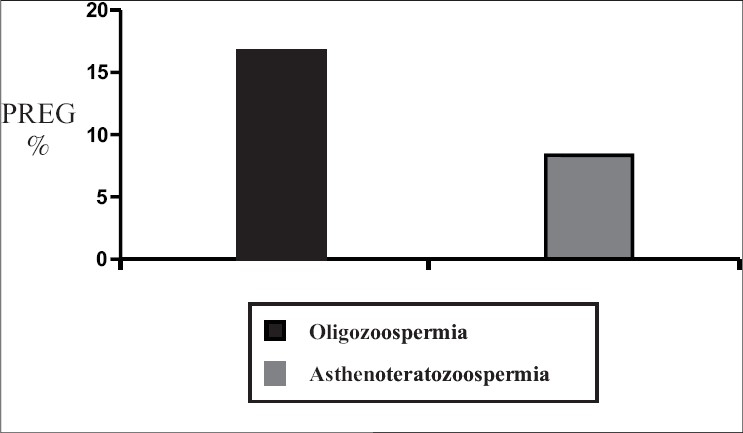
Intrauterine insemination outcome

**Figure 4:**
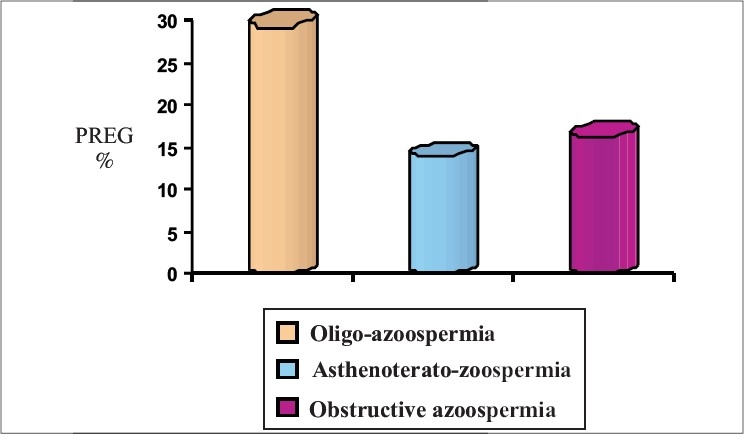
*In vitro* fertilization outcome

**Figure 5:**
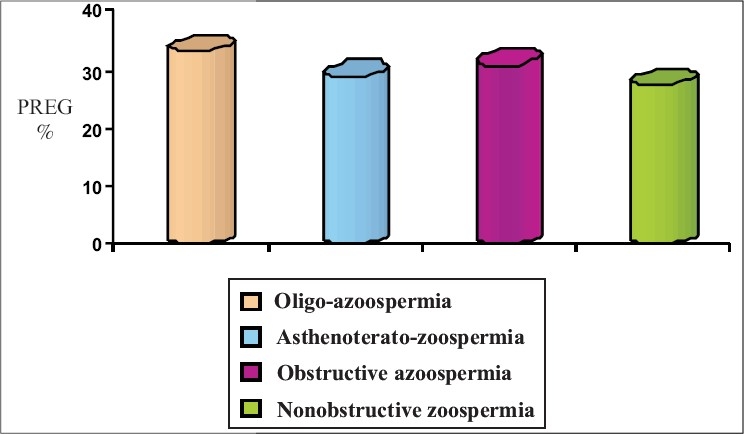
Intracytoplasmic sperm injection outcome

Oligozoospermia resulted in pregnancy rates of 16.66, 29.4 and 33.33% in IUI, IVF and ICSI respectively. Asthenoteratozoospermia had the lowest pregnancy rate (8%) with IUI, whereas IVF and ICSI resulted in pregnancy rates of 13.2 and 29% respectively. The two treatment modalities IVF and ICSI resulted in pregnancy rates of 16.36 and 30.9% respectively in obstructive azoospermia. In nonobstructive azoospermia, ICSI was the only treatment modality available that resulted in a pregnancy rate of 27.58% due to extremely poor recovery of mature spermatozoa [Table 2] [Figures 2–5].

**Table 2:** Assisted reproductive technology outcome in different male factor categories

	IUI	IVF			ICSI		
			
Group	Preg.%	Fertn%	Clev.%	Preg.%	Fertn%	Clev.%	Preg.%
Oligo. n = 153 (48, 51, 54)	16.66	70.98	81.74	29.4	75.48	87.11	33.33†
Asth.Terat. n = 158 (50,53,55)	8	30.77	63.33	13.2*	73.89	84.27	29*
Obstr.Azo. n = 110 (55, 55)	–	58.27	62.58	16.36	73.51	90.90	30.9*
Non-obstr. n = 58	–	–	–	–	52.16	73.31	27.58

* *P*, 0.0001

† *P*<0.005, IUI = Intrauterine insemination, IVF = *In vitro* fertilization, ICSI = Intracytoplasmic sperm injection

## DISCUSSION

Male factor-associated infertility has always been a challenge for infertility specialists, mainly due to its unpredictable nature and diversity. For many years, artificial insemination was the only treatment modality available for such cases, but resulted in poor outcome. Recent developments in the field of assisted reproductive technology have changed this scenario and newer techniques have enabled severe male factor patients to father their own biological children.

In terms of pregnancy outcomes, IUI resulted in a pregnancy rate of 16.66% in cases of oligozoospermia, which is quite acceptable and thus, IUI can be considered as the first line of treatment before switching over to IVF or ICSI. Although ICSI showed a significant increase in pregnancy rates as compared to IUI (*P* < 0.005), there was no significant difference between patients in the IVF (29.4%) and ICSI (33.33%) groups. This suggests that if the cause of infertility is oligozoospermia, there is no need for unnecessarily manipulating the female gamete for ICSI. If IUI is unsuccessful in 3–4 attempts, patients can be advised to go for IVF with efficacy equal to that of ICSI.

In case of asthenoteratozoospermia, ICSI (pregnancy rate of 29%) showed a clear advantage over IVF (pregnancy rate of 13.2%, *P* < 0.001) while IVF showed a clear advantage over IUI (pregnancy rate of 8%, *P* < 0.001). From these results for all the above three modalities, ICSI proved to be the most beneficial treatment modality for this group.

In obstructive azoospermia, sperms are obtained from the epididymis, where sperms are stored. Some researchers believe that epididymal sperms are not competent to fertilize the egg unless they pass through the ejaculatory ducts and come in contact with seminal vesicle and prostatic secretions.[8,9] However, there are some reports showing enough evidence for their compatibility of fertilization.[10,11] In spite of normal spermatogenesis, sperms could not be ejaculated into the semen due to either obstruction in the ejaculatory ducts or a congenital absence of the vas deferens. In this study, the sperms obtained from the epididymis were used for IVF and for ICSI. In IVF, the processed sperm were deposited in the vicinity of the oocytes in a droplet of culture medium. Sperms were retrieved from the epididymis, showing a statistically significant difference (*P <* 0.001) in pregnancy rates between IVF (16.36%) and ICSI (30.9%). One striking observation in this group was a rapid deterioration of sperm motility, which may the cause for poorer IVF outcomes.

ICSI was the only option available for patients with nonobstructive azospermia from whom sperms was extremely difficult to obtain. A decade ago, these patients did not have any hope to have their own children; today, ICSI has changed this situation. In the present study, a 52.16% fertilization rate with 73.31% cleavage and 27.58% pregnancy rates were obtained in this group, which was comparable with results published by other authors.[12,13]
